# Molecular detection (*k-ras*) of exfoliated tumour cells in the pelvis is a prognostic factor after resection of rectal cancer?

**DOI:** 10.1186/1471-2407-8-213

**Published:** 2008-07-27

**Authors:** Annette Torgunrud Kristensen, Johan N Wiig, Stein G Larsen, Karl-Erik Giercksky, Per O Ekstrøm

**Affiliations:** 1 Section for Surgical Oncology, Rikshospitalet-Radiumhospitalet Medical Center, Montebello, Oslo, Norway; 2 The Faculty of Medicine, University of Oslo, Norway

## Abstract

**Background:**

After total mesorectal excision (TME) for rectal cancer around 10% of patients develops local recurrences within the pelvis. One reason for recurrence might be spillage of cancer cells during surgery. This pilot study was conducted to investigate the incidence of remnant cancer cells in pelvic lavage after resection of rectal cancer. DNA from cells obtained by lavage, were analysed by denaturing capillary electrophoresis with respect to mutations in hotspots of the *k-ras *gene, which are frequently mutated in colorectal cancer.

**Results:**

Of the 237 rectal cancer patients analyzed, 19 had positive lavage fluid. There was a significant survival difference (p = 0.006) between patients with *k-ras *positive and negative lavage fluid.

**Conclusion:**

Patients with *k-ras *mutated cells in the lavage immediately after surgery have a reduced life expectation. Detection of exfoliated cells in the abdominal cavity may be a useful diagnostic tool to improve the staging and eventually characterize patients who may benefit from aggressive multimodal treatment of rectal cancer.

## Background

Cancer of the rectum is frequent in both genders and the incidence in Norway is still increasing, with a total of 1137 new cases in a population of 4.6 million citizens in 2004 [1]. Survival in cancer of rectum has continuously improved in the past ten year period [1,2]. The main reasons for this is the introduction of total mesorectal excision (TME) [3] and better staging with adjuvant treatment for patient in high risk groups [4]. In spite of such improvement, local recurrence after potentially curative surgery is a major problem. There are several possible causes for local recurrences including: incomplete resection of the primary carcinoma [4,5], insufficient removal of involved regional lymph nodes [6], development of a secondary tumour near the suture site and exfoliated cancer cells released at the time of surgery [7]. Dissemination of tumour cells in the presacral space, as well as in tumour lymphatic drainage and in peripheral blood vessels after resection of the tumour, might play a role in the metastasis process, thus affecting the clinical course. Previous reports have indicated that tumours can release DNA into the circulation [8-10]. Hence, recovery of mutated cell by pelvic lavage after resection of rectal cancer was hypothesized to be of prognostic value. One of the most prominent mutated genes in colorectal cancer is Kirsten ras 2 (*k-ras*), a member of the *RAS *family. Mutations in the *k-ras *gene is described as an early event in the process of colorectal carcinogenesis [11]. *K-ras *mutations are mainly found in exon 1, codon 12, 13 and exon 2, codon 61. Mutations in codon 12 account for 80–90% of *k-ras *gene alterations with several hotspots [12]. Oncogenic mutations of *k-ras *are involved in 20–50% of colorectal cancers [13,14]. The *k-ras *mutation frequency in rectal cancer has been reported to be 15–33% which is lower than in colon cancer [15,16]. We used *k-ras *exon 1 as the only tumour cell-associated marker to determine the presence of disseminated tumour cells in peritoneal lavage samples from patients undergoing surgery for rectal cancer. The aim of this pilot study was to evaluate the method denaturant capillary electrophoresis (DCE), to detect tumour cells in peritoneal lavage fluid of patients undergoing resection for rectal cancer.

## Methods

### Study population

The University Hospital Rikshospitalet-Radiumhospitalet is a tertiary referral center for locally advanced primary and recurrent rectal cancer. The treatment regime is multimodal and includes preoperative radiotherapy or chemoradiation.

Our patients undergo surgery a median 56 days after end of long course radiation therapy.

Peritoneal lavage from 237 patients and tumour samples from 186 patients (59% men, 41% women) with either locally advanced or local recurrent rectal cancer was collected during surgery at the Norwegian Radium Hospital between 2000 and 2006. Patient's age ranged from 29 to 87 years with a median age of 66. All samples were collected after written informed consent. Possible risk factors for dissemination of cancer cells intraoperatively were registered: resection through the periphery of the cancer as in R1 and R2 resections, tumour perforation or the presence of lymph node metastases in the mesorectum. Penetration of cancer through the serosa and the presence of macroscopic peritoneal carsinomatosis may of course give rise to free cancer cells and were thoroughly registrated. Two cases with registrated peritoneal carsinomatosis were tested as "positive controls". Tumours were pathologically staged according to the TNM classification [17] except for the local recurrences that do not fit into this except for pTo. This is due to the observation that a recurrence usually starts outside the rectal wall and most often there are no lymph nodes to resect [18]. The local surgical achievements were staged as follows: R0 microscopically free circumferential and distal margins, R1 microscopically involved margins, and R2 locally macroscopic residual cancer or no resection. N0; No regional lymph node metastasis. N1; Metastasis in 1 to 3 regional lymph nodes. N2; Metastasis in 4 or more regional lymph nodes. NX; unknown status of lymph nodes.

### Sample treatment

After resection of the tumour the pelvis was washed with sterile water 200–600 ml (discarded) subsequently followed by 200–600 ml saline water. Thereafter two specimens of 50 ml were aspirated to 50 ml centrifuge tubes. The second sample of 50 ml most often contained more white blood cells than the first sample because haemostasis was not complete. Cells were harvested by centrifugation at 1200 g for 10 minutes followed by removal of the supernatant. The cell pellets were frozen at -20°C until DNA extractions were performed. QIAamp DNA Kit (Qiagen, Valencia, California, USA) was used for the DNA extraction, following the manufacturers instructions.

### Polymerase chain reaction

The target sequences were amplified through a nested PCR protocol to circumvent amplification of a pseudogene. Primers were designed with use of Primer 3 [18,19]. The first PCR reaction consisted of approximately 50 ng genomic DNA, 0.05 U/μl *Taq*, polymerase, 0.005 U/μl PFU polymerase, 1× Buffer (ABgene), 2.5 mM MgCl_2_, and 0.4 mM dNTP mix (ABgene) and 0.15 μM of each primer (5'CTTAAGCGTCGATGGAGGAG3', 5'AGAATGGTCCTGCACCAGTAA3'). The second PCR used a 1:1000 dilution of the first PCR products as template while the polymerase, buffer, MgCl_2 _and dNTP concentrations stayed the same. Primers, one labelled primer with GC-clamp (5'- 6Fam-CGCCCGCCGCGCCCCGCGCCCGTCCCGCCGCCCCCGCCCGCCTCTATTGTTGGATCATATTC3') at a final concentration of 0.11 μM and one reveres primer (5'CATTATTTTTATTATAAGG3') in a final concentration 0.3 μM were added to the mix. The total volumes of both PCR reactions were adjusted to 10 μl with sterile water. Temperature cycling was performed in a Mastercycler^® ^(Eppendorf, Bergman AS, Lillestrøm, Norway) using the following cycling conditions: denaturation 5 min at 96°C, followed by 35 cycles of 95°C in 30 s, annealing 56°C in 30s (first PCR) and 48°C in 30s (second PCR) and 72°C in 60s.

We also analysed *k-ras *exon 2, and *braf *exon 11 and exon 15, but were unable to find any mutations with these markers in the lavage samples.

### Mutation detection

Amplified 6-fam labelled PCR products were analysed by denaturant capillary electrophoresis in a MegaBACE 1000 DNA Analysis System (GE Healthcare Bio-Sciences AB, Uppsala, Sweden). The base substitutions were separated by cycling temperature capillary electrophoresis (CTCE), with mean separating temperatures of 48.5°C and amplitudes of 3°C cycled 20 times. *K-ras *mutations in exon 1 were identified by co-analysis with a mutated internal standard in a similar manner as previously described by Bjørheim et al. [20]. Additionally, samples with a high mutant fraction were sequenced according to standard DNA sequencing protocol (GE Healthcare Bio-Sciences AB).

### Follow-up

The patients were followed for five years with X-ray of the chest and CT of the pelvis and abdomen every three to six months for five years or to the end of the study period. Data concerning death was crosschecked with the national health register.

### Statistics

Patient's data were prospectively registered in a database. All data was analyzed using the Statistical Package SPSS 12.5 for Windows. The chi-squared tests were used for statistical analysis and differences were considered significant at P < 0.05. For survival analysis, Kaplain-Meiers curves were constructed, and using the log-rank test assessed differences in survival between groups.

## Results

**Figure 1:**
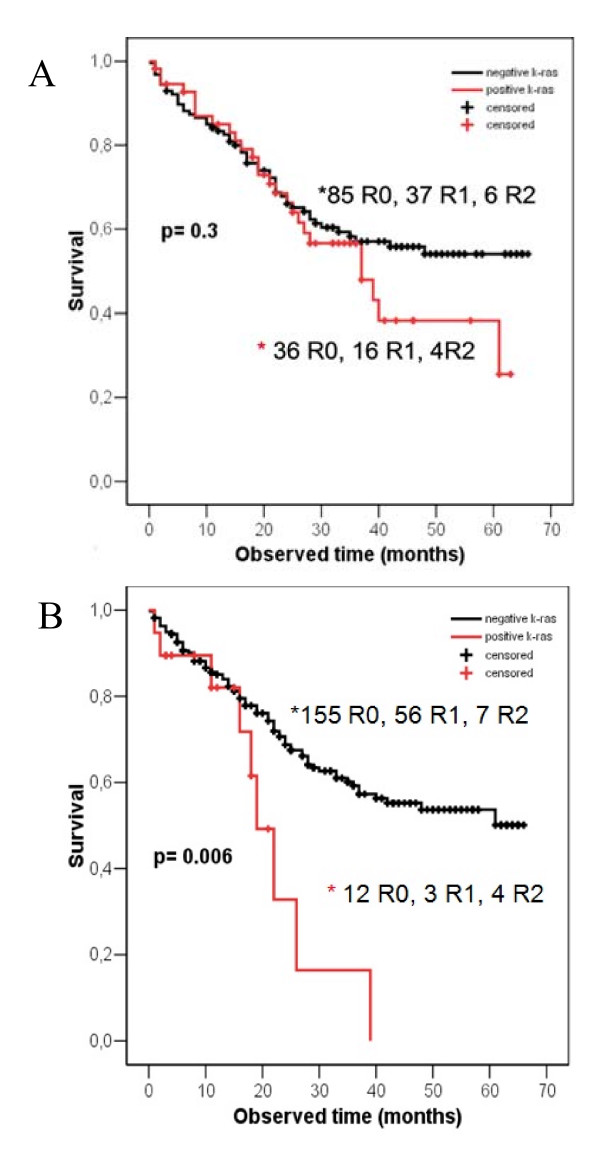
A. *K-ras *in lavage samples immediately after surgery related to survival. B. *K-ras *in tumours samples from rectal cancers patients related to survival.

The clinical pathological characteristics of the 237 rectal cancer patients are summarized in Table 1. Of the eleven possible risk factors tested with regard to patients with positive and negative *k-ras *markers in the lavage fluid, only N- and R-stage were significantly different between the two groups (p = 0.03 and p = 0.002, respectively). This was mainly due to a higher percentage of N0 and R0 stages in the *k-ras *negative group. We analyzed 186 of the removed tumours for *k-ras *mutations and 56 of these were *k-ras *positive. 19 out of 237 patients had a positive *k-ras *marker in the lavage fluid (Table 2). Of the 19 cases with *k-ras *positive lavage there were 12 R0, 3 R1 and 4 R2. We have estimated survival rate for the patients with positive and negative marker for lavage fluid by a Kaplan-Meier plot (Figure 1) and a log rank test. Mean observation time was 25 (1–66) months for all patients. Patients positive for the marker in the lavage fluid had a mean survival of 22 months with a Std. Error 3.7 (C.I 14.6–29.1) months compared to 47 months with a Std. Error 2.4 (C.I 43.2–50.8) for patients with a negative marker (p = 0.006). As R1 and R2 resections are more likely to have positive lavage fluid a new log rank test was done with only R0 patients. The result showed that there was still a significant difference between the two groups (p = 0.02).

**Table 1:** Clinical and pathologic parameters in patients with positive and negative *k-ras *mutations.

	k-ras marker Positive	k-ras marker Negative	P
Gender male/female	12/7	127/91	n.s

Age mean ± st.dv	65,8 ± 9.3	64.8 ± 11.8	n.s

Operation technique			

APR	7	88	n.s
LAR	6	79	
Hartmann's procedure	3	41	
EkspI.lap	2	4	
Tumour reduction	1	6	

Primary rectal cancer	13	176	n.s
Locally recurrent rectal cancer	6	42	

pT0-T1	0	19	n.s
pT2	2	21	
pT3	9	105	
pTx	8	47	

N0	6	125	p = 0.03
N1/N2	6	64	
NX	7	24	

M0	15	180	n.s
M1	4	38	

R0	12	155	p = 0.002
R1	3	56	
R2	4	7	

Distant metastasis during follow-up	4	70	n.s

Local recurrence during follow-up	7	31	n.s

Tumour perforation	5	37	n.s

APR: Abdominoperineal resection

LAR: Low anterior resection

EkspI. Lap: Explorative laparotomy

Local recurrence in this study means locally recurrent in the pelvis.

Distant metastasis in this study is distant metastasis or peritoneal carsinomatose.

**Table 2:** Clinical and pathologic information for the 19 patients with a positive *k-ras *marker in the lavage fluid.

Age	Gender	Operation tecnique	Sample status *	Recurrent^♣^	Tumour perforation	Metastatic	T	R	N	Serosa infiltration	Peretenoal carcino-matose	Time (months observed)	Status
69	M	LAR	primary	yes	yes	no	T3	R0	N0	no	no	26	Dead

64	M	APR	primary	no	no	no	T3	R0	N2	no	no	39	Dead

76	M	APR	primary	no	yes	no	T3	R0	N1	no	no	11	Dead

71	K	Hartmann	local recurrent	no	no	no	unknown	R0	Nx	yes	no	19	Dead

61	F	APR	local recurrent	no	no	no	unknown	R0	Nx	no	no	16	Dead

60	F	LAR	primary	no	no	no	T3	R0	N1	no	no	15	Alive

77	M	LAR	primary	no	no	no	T3	R0	N0	no	no	12	Alive

64	F	LAR	primary	no	no	no	T3	R0	N2	no	no	11	Alive

49	F	APR	primary	no	no	no	T3	R0	N2	no	no	4	Alive

60	M	APR	primary	no	no	yes	T2	R0	N1	no	no	8	Alive

68	M	LAR	primary	no	no	no	T2	R0	N0	no	no	3	Alive

71	M	APR	primary	no	no	no	T3	R0	N0	no	no	3	Alive

61	M	TumorRes	local recurrent	yes	yes	yes	unknown	R1	Nx	no	no	22	Dead

82	M	APR	local recurrent	no	no	no	T4	R1	Nx	no	no	18	Alive

59	F	LAR	primary	no	no	no	T3	R1	N0	possible	unsertain	3	Alive

46	K	Hartmann	primary	yes	yes	yes	T4	R2	N0	no	yes	21	Alive

66	M	EksplLap	primary	yes	unknown	yes	T4	R2	Nx	unknown	yes	1	Dead

69	M	EksplLap	local recurrent	yes	no	no	unknown	R2	unknown	no	no	18	Dead

78	M	Hartmann	local recurrent	yes	yes	no	T4	R2	Nx	no	no	2	Dead

APR: Abdominoperineal resection, LAR: Low anterior resection, EkspI. Lap: Explorative laparotomy

* Sample status describes when the patient's tumour is classified either as a primary or a local recurrence.

^♣ ^Recurrent describes whether or not the patients have developed a local recurrence after the surgery (when samples were retrieved).

There was no significant difference (p = 0.3) between *k-ras *positive and negative tumours with respect to survival (Figure 1b). Likewise when mutational status of *k-ras *for the tumour was compared for R0, the non-significant role was maintained (p = 0.09).

In two of the cases of R0 resections with *k-ras *positive lavage the preoperative biopsy was classified as *k-ras *negative being either a result of non-representative biopsy or a multi-clonal locally advanced tumour.

## Discussion

This study accessed the incidence of free colorectal cancer cells or remnants of such cells in the pelvic cavity immediately after surgery, and correlates their presence with several clinicopathological parameters and survival. A significant number of local recurrences following rectal cancer surgery cannot be explained by incomplete resection, lymphatic or vascular invasion [21]. This suggests that viable tumour cells with proliferate and perhaps metastatic potential have been shed from the primary tumour site either before removal of the tumour or during the surgical procedure. In our study we found that 19 rectal cancer patients had disseminated tumour cells in the 237 lavage fluids from the rectal patients. And we found that these patients with invisible tumour material in abdomen immediately after surgery tend to have a worse prognosis than patients without free tumour cells that could not be explained by differences in other risk factors. Only N- and R-stage were statistically significantly different between the *k-ras *positive and negative groups. In both cases this should be to the advantage of the *k-ras *negative group containing relatively more N0 and R0 stages.

Gross (R2) or microscopic (R1) positive surgical margins are well known indicators of recurrence and survival. However, our results showed that 12 of the 19 positive *k-ras *cases were R0 resections, which was an unpredicted result. Resection into the circumference of macroscopic (R2 resection) or microscopic tumour (R1 resection) or into the depth of the tumour can of course contribute to disseminated cells in the peritoneal cavity as well as is in cases with tumour penetration of serosa or in overt peritoneal carcinomatosis. As there were relatively more R0 stage resections in the *k-ras *negative group this is unlikely to explain our finding. Lymph node metastases might contribute by the shedding of cancer cells from a surgically severed lymphatic vessel. Several groups have observed a relationship between the number of lymph nodes identified at pathological examination and survival in colorectal cancer patients [22,23]. The higher percentage of N0 stage in the *k-ras *negative group could not explain the difference either. In our study there was no significant difference in estimated survival, but a possible trend observed between patients with *k-ras *positive and *k-ras *negative tumor markers in the solid tumor samples. This may rest upon the fact that the plots are more or less similar until 30 months postoperatively and diverge after this observation time. Thus within the observed period a *k-ras *mutation in the tumor itself does not explain the prognostic differences of the patients. In this study we have found that patients with tumour cells in abdomen after operation tend to have a worse prognosis than patients without free tumour cells. Our results in this study are similar to those of some studies with regard to survival [24-27], but in conflict with other results [28,29]. These differences could be related to patient selection and the methods used for detection as well as the follow-up methods.

It has become apparent in recent years that not only do the pathologic characteristics of rectal cancer influence the long-term outcome in terms of local recurrence and survival, but also that the surgeon is an important variable. This previously has been demonstrated by the reports from the Norwegian Rectal Cancer Group [30,31]. Better treatment, specialist care, recruitment into clinical trials or treatment protocols and any combination of these factors almost certainly influence regional variations in cancer outcome. The Norwegian Radium Hospital is a comprehensive cancer centre with high-volume surgery and the rectal cancers patient population is mostly TNM stage II and III. In this pilot study only rectal cancer patients have been included.

The detection of disseminated tumour cells depends on a number of steps including collection and treatment of samples, cell separation protocols and chosen markers. Technical advancements in detecting free DNA have been made over the years. All methods used depend on the recognition of antigens or gene markers assumed to be exclusively expressed by tumour cells and not by normal tissue in the examined sample. A cytology based method can only detect nanogram quantities of DNA. Immunohistochemistry leads to an increased sensitivity, but without improved specificity. High number of false positives due to non specific labelling was still observed and low sensitivity and specificity contribute to the conflicting results previously reported in detection of disseminated tumour cells [25,28,29]. With the introduction of PCR, smaller quantities of DNA could be detected. The robustness of such analysis has been improved by fluorescence-based allelotyping techniques involving capillary electrophoresis. These molecular detection methods can be divided in two groups: Detection of RNA with marker-gene expression (m-RNA) and detection of RNA specific to the tissue. This is a sensitive method with a possible detection of one single mutated cell in up to 10^8 ^wild-type cells in an optimal condition. However RNA is unstable in the extracellular environment and its detection in tissue or fluids is thus dependent on the presence of viable tumour cells. Routinely performed clinical sampling methods such as lavage may not be capable to preserve the RNA capacity of the cells. The other method is detection of tumour specific chromosomal abnormalities or mutations. In solid tumours these variations are heterogenous and complex with a need for detecting mutations in a small fraction. We have previously demonstrated that the DCE method used in this study fulfils these criteria with a sensitivity of 4 × 10^-3 ^[32].

The viability of exfoliated cells and the ability of these cells to become implanted and proliferated in the peritoneum have been confirmed by several studies [33,34]. We have not tested the viability of the tumour cells, because we assume that they are all compromised after the lavage treatment of sterilized water. A high diagnostic certainty is essential for establishing microscopic peritoneal dissemination as a prognostic factor. A standard procedure of peritoneal washing with sterile water subsequently followed by saline water was done after operation. Given the procedure and the sensitivity of the *k-ras *assay (limit is reported to be 1% for the homoduplexes and 0.1% for the heteroduplexes for a varity of target sequences), minor or moderate bleeding in the surgical area could result in a mutant fraction below the detection limit. One microliter of blood contains an average of 5000 white blood cells, hence if 10 millilitre of blood leaked into the lavage area, 50 000 *k-ras *positive tumour cells would be required for detection. Albeit this stringent limitation, *k-ras *mutant positive cells were regularly detected in lavage fluids from the pelvic cavity. There are no known tumour-specific markers for rectal cancer. *K-ras *is shown to be up-regulated in epithelial tumour cells [35] and was used as a marker for free tumour cells or cell remnants in the peritoneal cavity. The *k-ras *gene has a small hotspot region, which makes it possible to detect 80–90% of *k-ras *gene alterations with a simple PCR. However variation (20–50%) of the prevalence of *k-ras *mutations in small sporadic colorectal adenomas have been reported in the literature [35]. Specific *k-ras *mutations induce different biological consequences by affecting differently the structural confirmation and the function of the mutated protein [36,37]. For example, a number of studies have demonstrated an impact of *k-ras *mutations for cancer progression and predispose to more aggressive biological behaviour in patients with colorectal cancer [36,38-40]. *K-ras *mutations have also shown to be predictor of resistance to the anti-epidermal growth factor receptor (cetuximab) therapy and are associated with a an impaired prognosis [41].

In this study we found the frequency of *k-ras *mutations in tumour to be 30% and one-third of these showed *k-ras *mutations in the lavage fluid. Thus in 70% of these patients a *k-ras *mutant is absent. Therefore other gene mutations need to be investigated to obtain increased sensitivity. To date as mention earlier there is no general genetic marker that gives accurate information on the prognostic impact for patients with rectal cancer. New analyses of mutations or combinations of genes that are specific for rectal carcinoma might lead to better candidates. Detection of cancer specific markers in an abdomen without visible tumour deposits and with a proven increased risk of recurrence obviously imposes the question of additional therapy either as intraoperative hyperthermic chemotherapy or systemic chemotherapy or any combination of these. It should be remembered that this is a pilot study that needs to be confirmed in an appropriate multicenter study before therapeutic considerations are carried out.

## Conclusion

The overall clinical value of this molecular approach needs further elucidation. The *k-ras *gene seems to be a good genetic marker for detecting circulating tumour cells from rectal cancer patients. However, studies with gene markers covering a larger number of tumours are important. Examination of lavage fluids before and after surgery will further elucidate the mechanism of inducing exfoliated tumour cells. If our results are confirmed, in the future detection of disseminated cancer cells might enable the selection of high-risk patients with poorer prognosis, who would benefit from adjuvant treatment.

## Competing interests

The authors declare they have no competing interests.

## Authors' contributions

ATK participated in the design of the study, performed the experiments, data analyzes and drafted the manuscript. JNW and STG were responsible for all the samples from rectal cancer patient and clinical data, and revised the manuscript. KEG and POE conceived and participated in the design of study, revised the manuscript and supervised the whole process of the study. All the authors read and approved the final manuscript.

## Pre-publication history

The pre-publication history for this paper can be accessed here:


